# A Time Point Proteomic Analysis Reveals Protein Dynamics of *Plasmodium* Oocysts

**DOI:** 10.1016/j.mcpro.2024.100736

**Published:** 2024-02-10

**Authors:** Claude Marie François Preira, Elisabetta Pizzi, Federica Fratini, Felicia Grasso, Daniela Boccolini, Stefania Mochi, Guido Favia, Elena Piselli, Claudia Damiani, Inga Siden-Kiamos, Marta Ponzi, Chiara Currà

**Affiliations:** 1 Foundation for Research and Technology Hellas, Institute of Molecular biology and Biotechnology, Heraklion, Greece; 2 Core Facilities Technical-Scientific Service, Istituto Superiore di Sanità, Rome, Italy; 3 Department of Infectious diseases, Istituto Superiore di Sanità, Rome, Italy; 4 School of Biosciences & Veterinary Medicine, University of Camerino, Italy

**Keywords:** *Plasmodium*, malaria, oocyst, *Anopheles*, mosquito

## Abstract

The oocyst is a sporogonic stage of *Plasmodium* development that takes place in the mosquito midgut in about 2 weeks. The cyst is protected by a capsule of unknown composition, and little is known about oocyst biology. We carried out a proteomic analysis of oocyst samples isolated at early, mid, and late time points of development. Four biological replicates for each time point were analyzed, and almost 600 oocyst-specific candidates were identified. The analysis revealed that, in young oocysts, there is a strong activity of protein and DNA synthesis, whereas in mature oocysts, proteins involved in oocyst and sporozoite development, gliding motility, and invasion are mostly abundant. Among the proteins identified at early stages, 17 candidates are specific to young oocysts. Thirty-four candidates are common to oocyst and the merosome stages (sporozoite proteins excluded), sharing common features as replication and egress. Western blot and immunofluorescence analyses of selected candidates confirm the expression profile obtained by proteomic analysis.

## Highlights

• 581 oocyst proteins found.
• Expression varies while maturing.
• New antimalarial targets.

## Graphical Abstract

**Figure:**
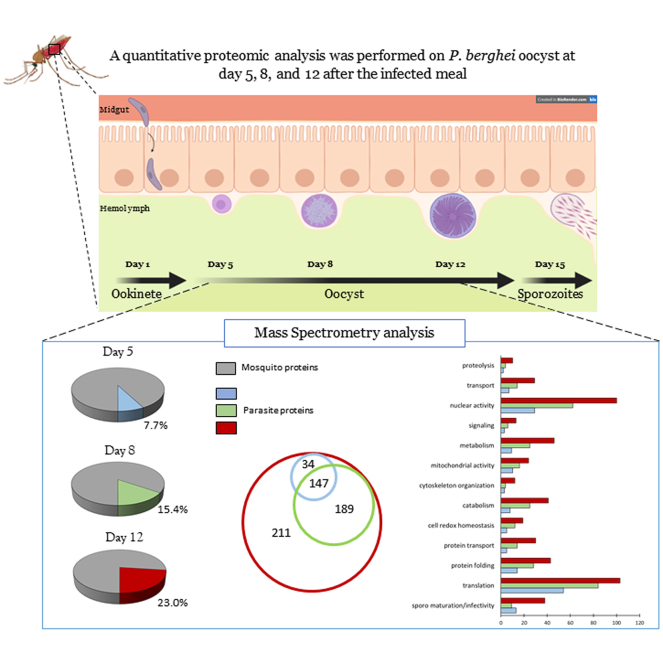

## In Brief

The purpose of this study is (i) to gain insights on molecules/pathways involved in *Plasmodium* oocyst maturation; (ii) to define the timing of expression and changes in protein abundance during oocyst maturation; and (iii) to define the topological characteristics of key biological processes by analyzing an oocyst-related protein–protein interaction network.

Malaria remains a devastating infectious disease causing 447 million new cases per year and 619,000 deaths in 2021 (World Health Organization malaria report 2022). The parasite is taken up by the mosquito when it takes a blood meal from an infected human. Once in the midgut of the mosquito, a complex series of developmental changes take place over the next roughly 3 weeks (for recent reviews, see Refs. (1, 2, 3)). The sexual phase including fertilization is initiated when gametocytes that have been formed in the blood are activated to mature gametes that fuse, and the resulting zygote develops into the motile ookinete. The ookinete is characterized by its elongated shape, microtubule cytoskeleton, and the micronemes, secretory organelles important for motility. About 24 h after the uptake of the infected blood meal, the ookinete will traverse the cells of the mosquito midgut epithelium until it reaches the basal lamina; here, it will undergo a reorganization of the cell to become the round and immotile oocyst where thousands of sporozoites form and mature in about 2 weeks. The early oocyst is characterized by massive protein synthesis concomitant with DNA replication, and ∼13 mitotic events take place. In the mature oocyst, the sporozoites are finally generated through budding; each sporozoite is assembled with a nucleus, mitochondrion, and the chloroplast as well as the characteristic secretory organelles micronemes and rhoptries (4). The sporozoites are characterized by their elongated shape; they are motile and use their complex motility machinery to traverse and invade cells in the mosquito and human. The mature sporozoites are released upon rupture of the wall of the oocyst, upon which they travel to and invade the mosquito salivary glands where they wait to be injected in humans’ through a new mosquito bite. While the ookinete and sporozoite stages have been investigated in detail (5, 6, 7), the oocyst in contrast is less well known as it is attached to the mosquito midgut and thus less amenable for study. An oocyst capsule of unknown composition protects the oocyst and separates it from the midgut tissue. The capsule is probably composed of mosquito-derived proteins, such as laminin, matrix metalloprotease 1 (MMP1), lysozyme c-1 (LYSC1) (8), and parasite-derived proteins. The *Plasmodium* capsule proteins identified so far are PbCap380 and PbCap93 (9, 10). Oocyst Rupture Protein 1 (ORP1) is also found at the capsule, where it interacts with ORP2, which is translocated to the oocyst periphery only in mature cysts (11, 12). The lack of either ORP1 or ORP2 affects oocyst rupture and consequently sporozoite egress.

All these capsule proteins were found to be essential for parasite survival in the mosquito. Another recently identified capsule protein, oocyst capsule protein (OSCP), is involved in ookinete motility and oocyst capsule formation. Parasites lacking OSCP showed reduced ookinete gliding motility and reduced number of oocysts (13).

Among the proteins with a key role in sporogonic stages is circumsporozoite (CSP), which localizes to the plasma membrane of the oocyst and is the major component of the sporozoite surface. This multifunctional protein is important for sporozoite formation and maturation, salivary glands targeting and invasion; lack of CSP also leads to failure of sporozoite development in oocysts (14). Nonetheless, CSP plays a role in sporozoite-hepatocyte contact and invasion (15) and during immune evasion strategies in the pre-erythrocyte stages (16). Thrombospondin-related protein 1 (TRP1), containing a thrombospondin repeat, is important for oocyst egress and salivary gland invasion by sporozoites. TRP1 is required for sporozoites to move prior to their exit from oocysts (17). Egress cysteine protease 1 has a crucial role in oocyst rupture, probably because of its enzymatic activity (18).

A limitation of understanding the biology of oocyst maturation and the mechanism of rupture is due to the difficulty of manipulating and isolating oocysts from the tissues of the mosquito to which they are attached.

Previous studies have focused on defining sporozoite proteomes of rodent and human *Plasmodium* parasites isolated from oocysts and salivary glands (7, 19, 20). However, only two studies included analysis of mature oocysts, identifying 127 (21) and 300 (22) proteins. It is important to note that no published data are available for younger oocysts.

Through quantitative proteomic and bioinformatic approaches, we here present the analysis of the oocyst proteome at three different time points, covering early, mid, and late stages of maturation. We show that key processes are tightly regulated and that the expression and abundance of oocyst-related proteins varies during oocyst maturation. This was also confirmed by immunolocalization assay for selected proteins. In addition, we defined, through a protein–protein interaction (PPI) network, the topological and functional relationships between the components of the mature oocyst. The present study is a pioneer in the study of *Plasmodium* oocyst components and opens new perspectives on the understanding of this poorly studied stage.

## Experimental Procedures

### Ethics Statement

All work was carried out in full conformity with Greek regulations and laws on animal experiments. In Greece, these issues are covered by the Presidential Decree (160/91) and law (2015/92), which implement the directive 86/609/EEC from the European Union and the European Convention for the protection of vertebrate animals used for experimental and other scientific purposes and the new legislation Presidential Decree 56/2013. The experiments were carried out in a certified animal facility, and the protocol has been approved by the FORTH Ethics Committee (101/14-12-2020) and by the Prefecture of Crete (license number #106323, April 29, 2021). All experimental procedures are done in accordance with ARRIVE guidelines.

#### Experimental Design and Statistical Rationale

The purpose of this study is (i) to gain insights on molecules/pathways involved in *Plasmodium* oocyst maturation; (ii) to define the timing of expression and changes in protein abundance during oocyst maturation; and (iii) to define the topological characteristics of key biological processes by analyzing an oocyst-related PPI network. Four independent groups of 20 oocyst-infected mosquito midguts were isolated at 5, 8, and 12 days after blood meal and analyzed separately by label-free quantitative mass spectrometry (MS) (Supplemental Table S1), each in three technical replicates. Reproducibility of protein abundances among biological replicates was assessed by ANOVA test (*p* < 0.0001; confidence 95%) on normalized abundance values (Supplemental Fig. S1).

#### Statistical Analysis

All statistical analyses were performed with XLSTAT Addinsoft 2023 (https://www.xlstat.com/en). PPI network of oocyst-related proteins was derived by mapping the 581 proteins identified at day 12 into the STRING database (https://string-db.org/). Filters were applied to exclude interactions predicted exclusively by text mining. Finally, we obtained a network consisting of 478 nodes and 16,420 edges. The main biological processes were mapped according to the functional annotation in Supplemental Table S2. Topological analysis of the network was performed by clustering and centrality analysis.

#### Parasite and Mosquito Maintenance

*Plasmodium berghei* ANKA 8417HP strain was used throughout the study (23) and was maintained in BALB/C mice (ENVIGO RMS SRL). Mice were male or female and ∼6 to 8 weeks when infected. *Anopheles stephensi* strain *Sinkasur* mosquitoes were reared as described (24). Mosquito infections were carried out by offering *A. stephensi* to mice infected with WT parasites.

Twelve different cages of adult mosquitoes were infected with *P. berghei* parasites, and midguts were collected at 5, 8, and 12 days post blood meal (p.b.m.). A total of 80 infected midguts (20 midguts per tube) were collected at each time point from different cages.

### Proteomic Analysis

Four biological replicates, obtained from independent feedings with different mice at different generations of mosquito, were analyzed for each time point (5, 8, and 12 p.b.m.). Protein extraction was performed by 3% sodium deoxycholate (SDC) in 20 mM triethylammonium bicarbonate (TEAB), and the concentration was assessed by BCA Protein Assay (Pierce Rapid Gold BCA Kit). Equal amounts (30 μg) from individual samples have been reduced (10 mM Tris(2-carboxyethyl)phosphine, 30 min at 56 °C) and alkylated (40 min iodoacetamide, room temperature in the dark) in solution. Protein mixture was then diluted to 450 μl in 8 M urea in 20 mM TEAB and transferred to Microcon-30 kDa Centrifugal Filter Unit (MERK) previously washed with 0.5% SDC in 8 M urea and 20 mM TEAB. After buffer exchange, trypsin digestion (sequencing grade–modified trypsin; Promega) was performed in 0.5% SDC in 20 mM TEAB, o.n. at 37 °C. Then 50 μl of 20 mM TEAB were added to the filter unit, and the tryptic digest was collected by centrifuging 5 min at 14,000*g*. The elution was repeated twice. To stop digestion and allow SDC to precipitate, 0.1% trifluoroacetic acid was added to a final concentration of 0.5%. A final extraction with 1:1 ethyl acetate was performed twice to eliminate any salt trace, and then the peptide solutions were lyophilized in speed-vac, resuspended in buffer A (5% acetonitrile and 0.1% formic acid), and quantified by Quantitative Colorimetric Peptide Assay (Pierce). For each biological replicate, 3 μg were analyzed in technical triplicate using an Orbitrap Fusion Tribrid Mass Spectrometer (Thermo Fisher Scientific) coupled to an UltiMate 3000 UHPLC system (Thermo Fisher Scientific). Peptides were firstly trapped in a μ-precolumn (C18 PepMap100, 5 μm, 100 Å, 5 mm × 300 μm; Thermo Fisher Scientific) and then run on a home-packed 50 cm × 75 μm id fused-silica column (8 PicoTip Emitter; New Objective) packed with ReproSil-Pul C18-AQ 1.9 um beads (Dr Maisch GmbH) for chromatographic separation. Peptides were eluted at 0.2 μl/min along a 180 min linear gradient from 7% to 30% of buffer B (95% acetonitrile and 0.1% formic acid). Columns were heated at 42 °C. Full-scan MS was acquired in Orbitrap at 60 K resolution—maximum injection time of 50 ms, one microscan, wide quadrupole isolation activated in a mass range of 350 to 1550, and an automatic gain control target of 4E5. The MS/MS scans were automatically acquired in the ion trap for a total cycle time of 3 s; quadrupole isolation window 1.6; minimum intensity 5E3; high-energy collisional dissociation fragmentation; NGC 32; normal scan rate; maximum injection time of 35 ms; and automatic gain control of 5E3. Dynamic exclusion allowed a repeat count of 1 within 45 s; maximum tolerance of 10 ppm. Spectra raw files have been processed by Proteome Discoverer 2.3 (ThermoFisher Scientific). Processing workflow for precursor-based quantification. Ion trap-detected high-energy collisional dissociation spectra using SequestHT with Percolator validation. A combined database containing PlasmoDB-46_PbergheiANKA, National Center for Biotechnology Information *Anopheles_Stephensi*_2020 and common contaminants followed by concatenated decoy database was used as protein database. Trypsin (full) digestion; two maximum missed cleavage sites. Precursor mass tolerance: 10 ppm; fragment mass tolerance: 0.5 da. Dynamic modification: oxidation of methionine/+15.995 Da and N-term acetylation/+42.011 Da; static modification: carbamidomethyl of cysteines/+57.021 Da. High confidence was selected, meaning false discovery rate set at 0.002 for peptide-spectrum matches; 0.009 for peptide groups and 0.010 for proteins. Database file contains 33,960 entries (50% decoy).

#### Immunofluorescence Assay

Midguts were dissected at different days after the blood meal and fixed for 1 h in 4% paraformaldehyde/1% saponin in PBS. In the following, all incubations were carried out for 1 h at room temperature. After three washes in PBS/1% saponin, parasites were blocked with 3% bovine serum fraction/PBS, incubated with the primary antibody diluted in PBS/1% saponin, washed several times in PBS, after which the secondary antibodies were added (1:800 dilution). Cell nuclei were labeled with 4,6-diamidino-2-phenylindole. Samples were viewed using a Zeiss Axioskop 2 plus microscope and, in some cases, analyzed using LEICA SP8 confocal laser scanning microscope, and images were displayed and analyzed using the LAS X 3.5.7.23225 software (Leica Microsystems). Images were further processed using ImageJ (Natioanl Institutes of Health - NIH). Immunofluorescence assay was repeated at least three times for each antibody.

#### Antibodies

The ookinete surface protein antibody anti-P28 clone 13.1 (25), enolase antiserum (26), 14.3.3 polyclonal rabbit immune serum (27), and *P. berghei* PSOP1 polyclonal mouse immune serum (28) were diluted 1:100 in immunofluorescence assay and 1:1000 in Western blot analysis.

The monoclonal antibody recognizing CSP (clone 3D11) described (29) and the *P. Berghei* Cap380 (9) were diluted 1:500 in immunofluorescence assay and 1:5000 in Western blot analysis.

The monoclonal P25 antibody described (30) was used 1:25 in immunofluorescence assay and 1:500 in Western blot.

The secondary antibodies conjugated with Alexa Fluor were purchased from ThermoFisher Scientific; Alexa-Fluor 555 goat antimouse IgG (catalog number: A21424) and Alexa-Fluor 488 goat anti-rabbit IgG (catalog number: A11034), and they were all diluted 1:800.

Immune sera were used in the present work diluted 1:100 in immunofluorescence assay and 1:1000 in Western blot assay.

#### Western Blot Assay

Protein extracts from adult mosquito midguts infected with *P. berghei* parasites were separated on 12% SDS polyacrylamide gel and transferred to a nitrocellulose membrane (GE Healthcare) using MINI TRANS-BLOT (Bio-Rad). After blocking overnight with 5% nonfat dry milk in PBS with 0.1% Tween-20, membranes were incubated with primary specific antibodies, followed by incubation with antimouse horseradish peroxidase–conjugated secondary antibody (1:10,000 dilution). The immunocomplexes were visualized using chemiluminescence ECL detection system (Luminata Forte Western HRP Substrate; Millipore) according to the manufacturer's instructions. The secondary antimouse and anti-rabbit antibodies conjugated with horseradish peroxidase, used for Western blot assay, were all diluted 1:10,000.

## Results

### Quantitative Analysis of Oocyst Proteome During Sporozoite Maturation

*P. berghei*-infected midguts were hand dissected at 5, 8, and 12 days after the blood meal, each in four biological replicates (20 per replicate), and subjected to quantitative MS. In total, we identified 581 parasite-specific proteins with at least two unique peptides: a maximum of 181 parasite proteins at day 5; 354 at day 8, and 581 at day 12, with an overlap between biological replicates >90%. These proteins accounted for 7.7%, 15.4%, and 23% of total identifications at days 5, 8, and 12, respectively (Supplemental Table S1), because of the contamination of mosquito midgut proteins on which oocysts are anchored.

**Fig. 1:**
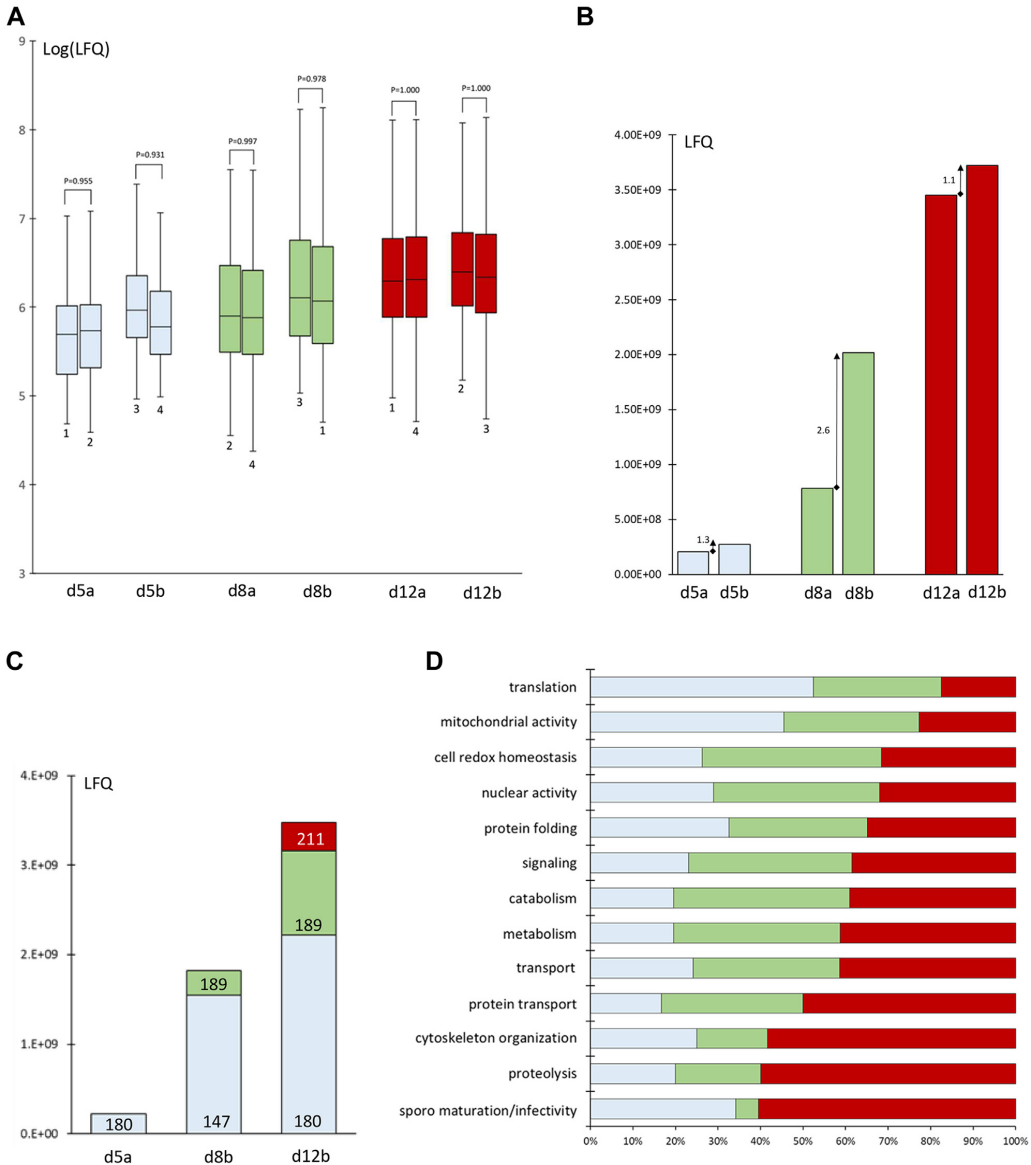
**Analysis of oocyst proteome at different time points.** Label-free quantitative (LFQ) proteomics on isolated *Plasmodium berghei*–infected mosquito midguts was performed in four biological replicates at day 5 (d5), day 8 (d8), and day 12 (d12) after blood meal. *A*, at each time point, pairs of replicates with the highest correlation values were grouped, day 5: d5a (replicates 1 and 2) and d5b (replicates 3 and 4); day 8: d8a (replicates 2 and 4) and d8b (replicates 1 and 3); and day 12: d12a (replicates 1 and 4) and d12b (replicates 2 and 3). Box plots of log LFQ values (normalized LFQ) shows a normal distribution and similar mean values between replicates of each subgroup. *t* Test probability values (two-tailed paired; alpha = 0.05) are indicated. *B*, the total abundances, calculated per each subgroup (d5a = 2.09 × 10^8^, d5b = 2.76 × 10^8^; d8a = 7.83 × 10^8^, d8b = 2.02 × 10^9^; d12a = 3.45 × 10^9^ and d12b = 3.72 × 10^9^), were represented as histograms. The fold change in abundance between subgroups is indicated. *C*, a stacked bar chart representing total LFQ of a group of 180 proteins that start to be detected at day 5 (*light gray*) and a group of 189 proteins that start to be detected at day 8 (*green*). About 211 additional proteins were exclusively detected at day 12 (*red*). *D*, the identified proteins were classified based on the functional annotation; the percentage of proteins belonging to the different functional categories at each time point are shown.

When we analyzed quantitative data (ANOVA, confidence interval 95%, tolerance 10^−3^, Supplemental Fig. S1), protein abundances at day 5 and 8, varies between replicates, defining two, clearly distinct, subgroups: d5a (replicates 1 and 2, *R* = 0.92) and d5b (replicates 3 and 4, *R* = 0.85); d8a (replicates 2 and 4, *R* = 0.98) and d8b (replicates 1 and 3, *R* = 0.97). At difference, when we considered pairs of replicates at day 12 oocysts, the protein abundances were similar (*R* ≥ 0.93), although two subgroups could be identified: d12a, replicates 1 and 4 (*R* = 0.99) and d12b (replicates 2 and 3, *R* = 0.94). The normalized replicates of each selected subgroup had a normal distribution and similar means of abundances, as verified by *t* test (Fig. 1*A*). The protein Cap380 (PBANKA_1218100) was excluded from the analyses because it accounted for 24.6% of the total protein abundance at day 5.

The presence of two distinct subgroups in early oocysts can be explained by the fact that the 80 infected midguts (20 per biological replicate) were hand dissected in 6 to 8 h. It is conceivable that this time frame may preferentially affect young oocysts undergoing active protein synthesis rather than 12 day oocysts, in which sporozoite maturation is almost complete.

As shown in Figure 1*B*, total protein abundances differ between subgroups of the same time point, and the highest difference value (2.6-fold increase) was observed at day 8, suggesting a peak in protein synthesis at this time point, whereas a less pronounced difference was found at day 5 and 12 (1.3 and 1.1, respectively).

To investigate how protein abundance is modulated during oocyst maturation, we selected three subgroups for comparative analyses: 5a (5b with a correlation value less than 0.9 was discarded) and the subgroups 8b and 12b with the highest relative abundance. We considered two subsets of proteins: a group of 180 proteins detected in day 5 oocysts and a group of 189 proteins detected in day 8 oocysts (Fig. 1*C*). These subsets were also identified in the oocyst proteome at day 12, which includes 211 additional proteins (Fig. 1*C*). The day 5 protein group increases progressively in abundance during oocyst maturation, peaking at day 8 (7-fold increase), whereas the day 8 subset increases only twofold at day 12. This indicates a nonlinear relationship in the increase in abundance between the time points and confirms a peak in protein synthesis at the transition between oocysts at days 5 and 8.

### Oocyst Functional Processes are Highly Coordinated

We then assigned a functional category to the identified proteins based on Gene Ontology annotation, reviewed manually (Supplemental Table S2), and calculated the percentage of proteins that fell into the different categories at each time point. We observed that several categories are represented differently during oocyst maturation (Fig. 1*D*). For example, about 50% of proteins involved in the translation process or mitochondrial activity had already been identified in the proteome of 5-day-old oocysts, whereas about 60% of the proteins involved in cytoskeleton organization, proteolysis, or sporozoite maturation/infectivity were identified in the proteome of 12-day-old oocysts.

In the latter category, we included proteins based on literature evidence or comparative analysis of available proteomes of blood and mosquito stage parasites of both *P. berghei* and *Plasmodium falciparum* (Supplemental Table S2). Eleven proteins, detected in our study, had previously been identified exclusively in sporozoite proteomes, including three components of the inner membrane complex (PBANKA_0402600, PBANKA_1311300, and PBANKA_1354900) and the putative G2 protein (PBANKA_0830400) involved in gliding motility. In addition to the well-characterized capsule protein Cap380 (2), five identified proteins were classified as being involved in oocyst formation, based on a comparative analysis of available *Plasmodium* proteomes, namely PBANKA_0711700 and PBANKA_1227900 identified in our oocyst proteome and merosomes of *P. berghei* but not in sporozoite proteomes of both *Plasmodium* species; PBANKA_0618800, PBANKA_1226700, and PBANKA_1452200 detected only in this study.

Collectively, when we considered the quantitative data, each functional category increases significantly in relative abundance by day 12, except for the translation process, which peaks at day 8 (Supplemental Fig. S2*A*).

By breaking down the assigned functional categories (Supplemental Table S2 and Supplemental Fig. S2*B*) into specific pathways, we observed a modulation of abundance between time points. For example, in the case of biogenesis and ribosome assembly, the peak is reached at day 8, whereas proteins involved in replication and gliding motility are found almost exclusively at day 12.

The presence of transmembrane (TM) domains or signal peptides (SPs) (Supplemental Table S2) was predicted by dedicated tools in the PlasmoDB database: 77 proteins have one or more TMs, 38 an SP, and 21 both, representing collectively the 23.9% of the proteins belonging to the major functional categories (Supplemental Fig. S3). As shown by pie charts in Supplemental Fig. S3, TM-containing proteins are most represented in the transport, protein transport and mitochondrial activities categories, whereas 28.9% of SP-containing proteins fall into sporozoite maturation/infectivity and 23.68% into protein folding; interestingly, 42.86% of proteins containing both TM and SP belong to the sporozoite maturation/infectivity category.

### Functionally Related Proteins are Topologically Close to Each Other

**Fig. 2:**
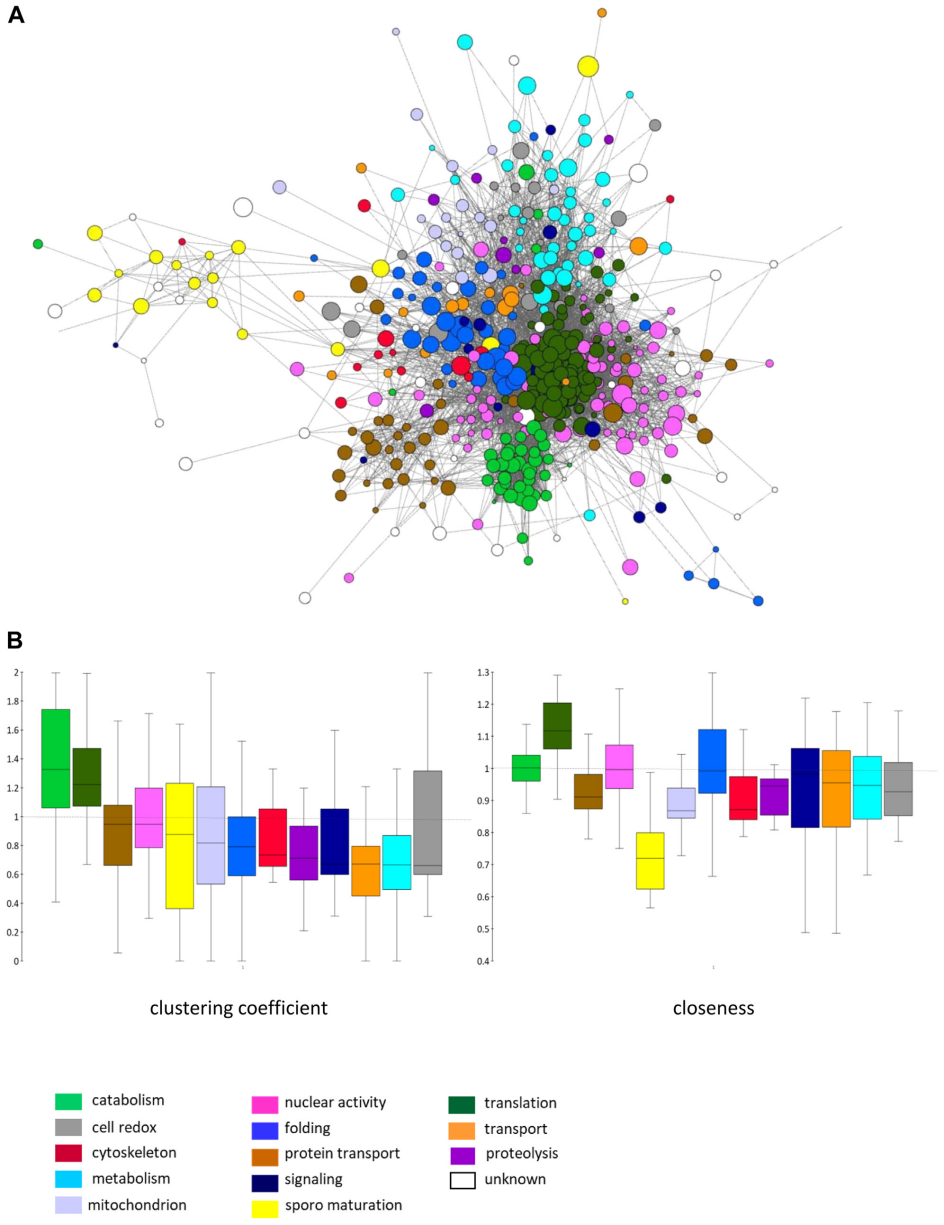
**Oocyst protein–protein interaction (PPI) network.***A*, graphical representation of the PPI network of mature 12-day-old oocysts extracted from the STRING database and visualized by GeneMania layout in the CytoScape platform. Nodes are colored according to the functional annotation of the corresponding proteins. *B*, box plot showing the distributions and means of centrality measures (clustering coefficient and closeness) reported for each functional group (*dashed line* indicates the network mean value).

To define the physical/functional relationships among the proteins expressed in mature oocysts, a PPI network was generated for the proteins identified at day 12 and visualized with the GeneMania layout to highlight functionally related groups of proteins (Fig. 2*A*). Visual inspection of the graph reveals the overall modular PPI architecture of the oocysts. In particular, the central location of proteins involved in key biological processes, such as translation, catabolism, nuclear activity, and the peripheral location of proteins involved in sporozoite maturation. These observations were confirmed by centrality analysis (Fig. 2*B*), calculating the clustering coefficient and closeness for each group of functionally related proteins. Proteins annotated as involved in catabolism and translation show high values of both indices, suggesting that these proteins tend to form highly interconnected clusters in the central region of the network. In contrast, stage-specific proteins involved in oocyst maturation and sporozoite development have mean of clustering coefficients close to that of the network but low values of closeness, indicating a peripheral location of the main cluster they form.

**Fig. 3:**
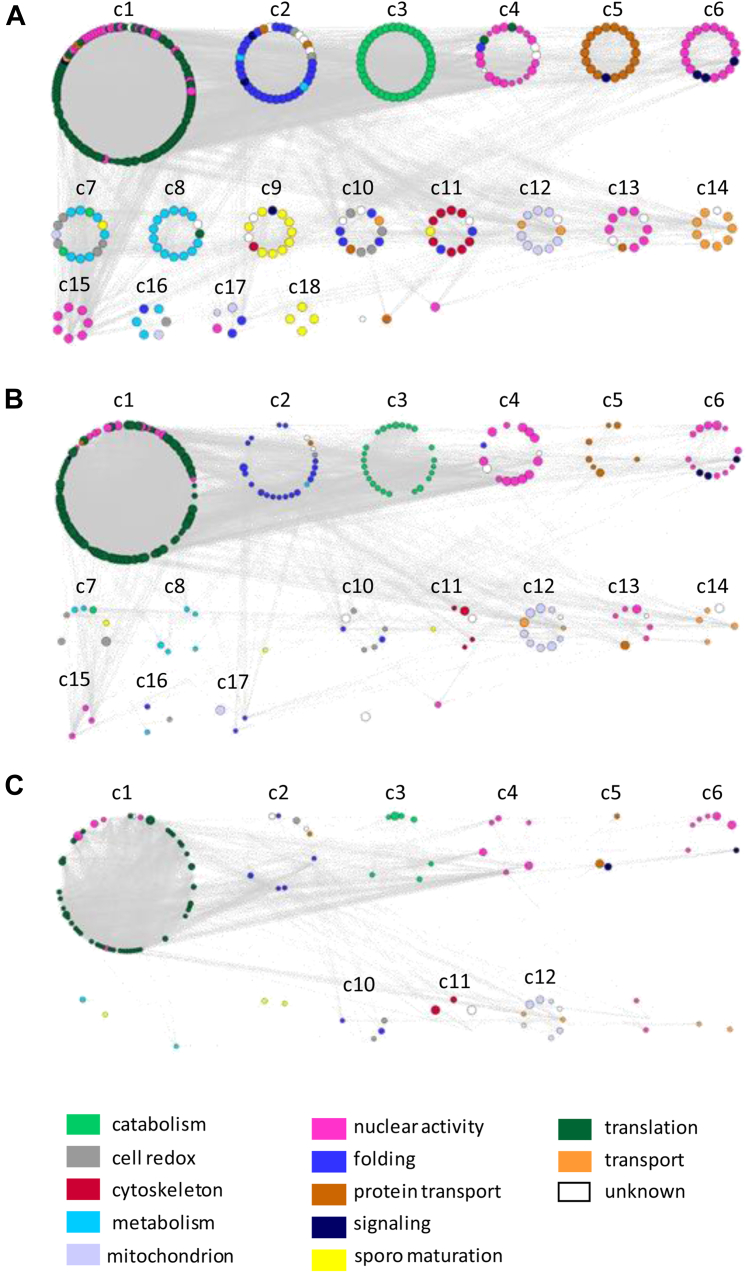
**Protein–protein interaction (PPI) network analysis.***A*, cluster analysis of the PPI network of the 12 day oocysts, performed with the Markov clustering (MCL) algorithm (granularity 2.5), identifies 18 topological clusters with at least four nodes that in most cases overlap with functional categories. Only a few clusters are populated when proteins detected at day 8 (*B*) and 5 (*C*) were mapped.

Cluster analysis performed by Markov clustering algorithm (granularity 2.5) confirms that functionally related proteins are topologically close to each other (Fig. 3*A* and Supplemental Table S2).

These spatially/functionally related groups of proteins detected in day 12 oocysts are almost absent in day 5 oocysts, where only the translation-related cluster is evident, and partially populated in day 8 oocysts (Fig. 3, *B* and *C*).

Overall, these results suggest close spatiotemporal coordination of oocyst processes leading to the maturation and emergence of sporozoites.

### Protein Expression and Localization During Oocyst Maturation

**Fig. 4:**
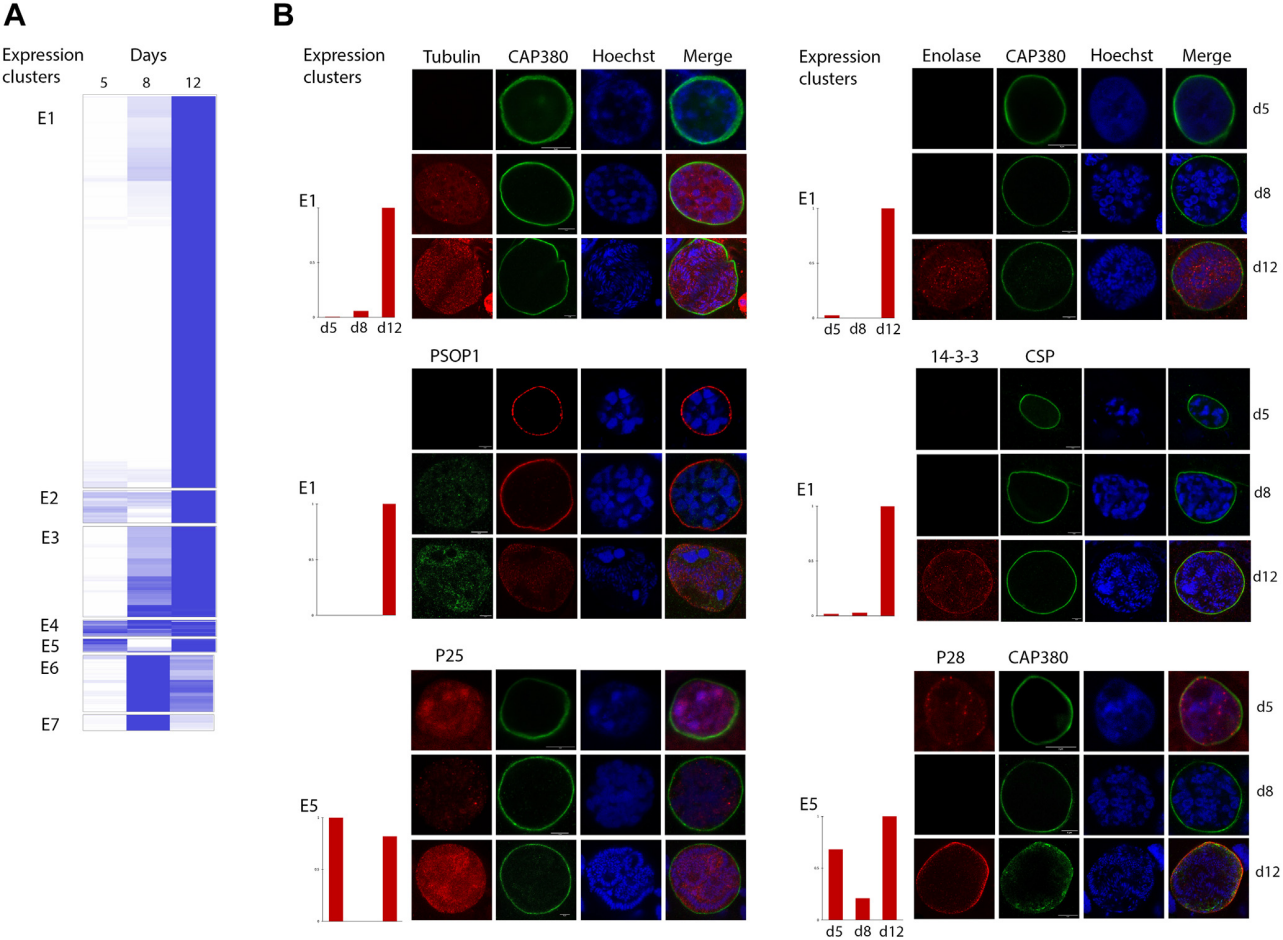
**Protein expression and localization through the oocyst time point development of selected candidates.***A*, cluster analysis of protein expression at day 5, 8, and 12 p.b.m. and related (clusters E1–E7) depicted as a heat map. *B*, immunofluorescence assay on wildtype oocysts at the selected time points (5, 8, and 12 days p.b.m.) using specific sera or antibodies recognizing selected candidates showing the localization and abundance during oocyst development and relative cluster of expression. According to the primary candidate antibody (mouse or rabbit), PbCap380 was used as a capsule marker or CSP as a marker of the plasma membrane. All nuclei were stained with Hoechst. Scale bar represents 5 μm. CSP, circumsporozoite; p.b.m., post blood meal.

Proteomic analysis suggests that the expression and abundance of proteins involved in oocyst-related processes vary in a coordinate manner during oocyst maturation. This is also evident from the clustering of the abundance profiles of the detected proteins, which can be separated into seven distinct groups (Fig. 4*A*). For example, cluster E1 includes proteins detected mainly at day 12, whereas cluster E6 proteins whose abundance peaks at day 8; cluster 5 includes proteins expressed at day 5 and 12 but probably switched off at day 8. To further confirm this observation, we selected candidate proteins from different clusters to follow their expression and subcellular localization during oocyst development. Analysis was limited to six proteins for which antibodies were available. Western blot analysis confirmed that the selected proteins were all expressed in late oocysts (Supplemental Fig. S4). Next, we performed immunolabeling experiments on oocysts from midguts dissected at the three different time points. In Figure 4*B*, we show that the expression and localization of the selected proteins in early, mid, and late oocysts is consistent with their proteomic profile when analyzed by confocal microscopy in colabeling with capsule-specific Cap380 or membrane CSP.

P28 and P25 are ookinete surface proteins important for the development of this stage (31). Interestingly, P25 and P28, both in cluster 5, show similar expression pattern but different cellular localization in the developing oocyst. For both proteins, the strongest signal is observed at day 12, when sporozoites are formed inside the cyst, and the two proteins clearly localize in distinct cellular compartments. P25 remains inside the oocyst, whereas P28 colocalize with the capsule protein Cap380.

Cytoskeletal protein beta-tubulin and enolase (cluster 1) are mainly detected in mature oocysts at day 12, when sporozoites are forming. In particular, enolase-specific signal also decorates the periphery of oocyst. Interestingly, it has been previously shown (32) that in ookinetes enolase localizes to the parasite periphery to promote invasion of mosquito midgut. It is conceivable that the increased expression of enolase in late oocysts may be related to the function of invasive sporozoites.

*Plasmodium* 14-3-3 belongs to a family of proteins with multitask cellular functions such as signal transduction or protein trafficking. The 14-3-3 expression profile has been characterized in *P. berghei* and *P. falciparum* asexual and sexual blood stages (27). In this study, we show that Pb14-3-3 is mainly detected in the cytoplasm of day 12 oocysts (cluster 1) consistent with proteomic data.

PSOP1 is a secreted protein detected in the osmiophilic bodies of *P. berghei* (28) and *P. falciparum* (33) gametocytes and in the micronemes of *P. berghei* and *Plasmodium yoelii* ookinetes (34). Here, we show that PSOP1 is also expressed in late oocysts (cluster 1), where it localizes to the cyst capsule.

## Discussion

Despite that the oocyst stage is the longest of all *Plasmodium* life cycle, the oocyst formation and maturation is poorly studied. Although this stage offers an open window of about 2 weeks for targeting with antimalarial strategies, to date, a limited number of oocyst-related proteins have been identified (21, 22). In this study, through a quantitative proteomic approach, we have increased the number of oocyst-related molecules identified and, pioneering this field, defined changes in protein expression profile and abundance during oocyst maturation. The major functional categories are represented differently in early, mid, and late oocysts. For example, we showed that components of the translational apparatus or processes related to mitochondrial activity are major components of early oocysts, whereas proteins/pathways involved in DNA replication or sporozoite infectivity and motility were mainly detected in late oocysts, suggesting that the processes leading to oocyst maturation and sporozoite egress are tightly regulated temporally.

The relationships between proteins were studied through the analysis of a PPI network derived from the proteome of late oocysts. Functionally related proteins are spatially clustered revealing a modular organization of the network. General processes, such as translation or nuclear activity, are highly interconnected and occupy the central region of the network, whereas processes related to oocyst and sporozoite maturation are located at the periphery of the network.

We also compared the proteome of *P*. *berghei* at the oocyst stage with that of the available sporozoites and blood stages of both *P. berghei* and *P. falciparum*. This analysis revealed that 12 oocyst proteins were also detected in the proteome of isolated sporozoites, whereas three, namely PBANKA_1226700, expressed in early, mid, and late oocysts, PBANKA_1452200 and PBANKA_0618800 expressed only in late oocysts, were not detected in the proteome of sporozoites or other stages of the parasite, thus suggesting their specific structural/functional role in oocyst maturation. All these features could be exploited to identify new candidate proteins possibly involved in key processes for parasite transmission.

Interestingly, our oocyst proteome also includes proteins specifically detected in the proteome of schizonts or merosomes. Although the cellular contexts are different, we speculate that proteins involved in sporogony in the oocyst may also play a role in other *Plasmodium* multiplication stages.

Among the oocyst proteins identified in other invasive stages is PBANKA_1309600, a conserved *Plasmodium* membrane protein, which has been shown to play a role in sporozoite formation within oocysts. The mutant lacking PBANKA_1309600 (RMgm database) produces normal numbers of oocysts; however, sporozoite formation within these oocysts was not detected (21). The early function of this protein in sporozoite development is in agreement with its presence in oocyst-derived sporozoites and absence in salivary gland sporozoites. A BRO1 domain–containing protein, PBANKA_1439100, identified in the proteomes of schizonts and sporozoites of *P. falciparum* was shown to be important for oocyst maturation (35), in accordance with our data.

Although a mechanism of gliding motility and the presence of an inner membrane complex are maintained in different parasite stages, we identified components specific to mosquito stages. In particular, the putative G2 protein, PBANKA_0830400, is important for both ookinete and sporozoite motility (36). Mutant parasites lacking G2 protein showed ookinetes with aberrant morphology. Mosquitoes infected with G2-KO parasites formed oocysts that developed normally (albeit in reduced numbers) and formed a large number of abnormally shaped sporozoites that were unable to invade salivary glands (37). In addition, three inner membrane complex proteins, PBANKA_0402600, PBANKA_1354900, and PBANKA_1311300, with a peak of expression in mature oocysts were identified only in sporozoites.

As noted previously, the biological processes leading to oocyst maturation and subsequent rupture with release of the sporozoites into mosquito hemocoel are temporally regulated. By cluster analysis of quantitative proteomic data, we grouped the expressed proteins into seven clusters based on their abundance in early, mid, and late oocysts. These expression profiles were also confirmed for a limited number of proteins by confocal microscopy using specific antibodies.

Interestingly, among the proteins that we analyzed further are the ookinete surface proteins P25 and P28, which are expressed in early (day 5) and late oocysts (day 12) but not in day 8 oocysts. The downregulation of these proteins at day 8 oocysts is consistent with previous findings (9), which showed that major ookinete surface proteins such as P25 and P28 persist in early oocysts but are not detectable at day 6 of oocyst development.

Remarkably, re-expression of P25 and P28 was observed in mature oocysts, when sporozoite formation is complete. P25 was detected internal to the oocyst, but it is not possible to define whether its localization is specific to sporozoite or to oocyst compartments, whereas P28 localizes to the surface of the capsule, supporting the idea that the protein is a candidate for transmission-blocking strategies in oocysts, as suggested for the ookinete stage (38).

In conclusion, our approach to studying the oocyst stage by comparative quantitative MS has enabled us to identify new proteins with potential function in vector stages. Given the concordance of our results with the limited number of proteins described in the literature, the availability of the oocyst proteome at different time points may contribute to elucidate peculiar pathways of this mosquito stage, thus opening new possibilities for exploring this poorly studied *Plasmodium* stage and identifying new potential candidates for developing strategies to block malaria transmission.

## Data Availability

Proteomic raw data have been submitted to massIVE MS data repository. Link for dataset direct URL: ftp://MSV000091377@massive.ucsd.edu. Login details: reviewer ID is MSV000091377_reviewer; password is 00cy5.

## Supplemental data

This article contains supplemental data.

## Conflict of interest

The authors declare no competing interests.
