# Supplementary material for: A Time Point Proteomic Analysis Reveals Protein Dynamics of Plasmodium Oocysts
Source: Mol Cell Proteomics. 2024 Feb 10;23(3):100736. doi: 10.1016/j.mcpro.2024.100736 (PMC10924140; doi:10.1016/j.mcpro.2024.100736)
Supplement: Supplemental Figures [file mmc1.pdf]

A)

	day5_1	day5_2	day5_3	day5_4
day5_1	**1**	0.920	0.541	0.402
day5_2		**1**	0.646	0.398
day5_3			**1**	0.848
day5_4				**1**

	day8_1	day8_2	day8_3	day8_4
day8_1	1	0.470	0.984	0.508
day8_2		1.000	0.453	0.968
day8_3			1.000	0.503
day8_4				1.000

	day12_1	day12_2	day12_3	day12_4
day12_1	1.000	0.949	0.957	0.988
day12_2		1.000	0.939	0.929
day12_3			1.000	0.959
day12_4				1.000

B)

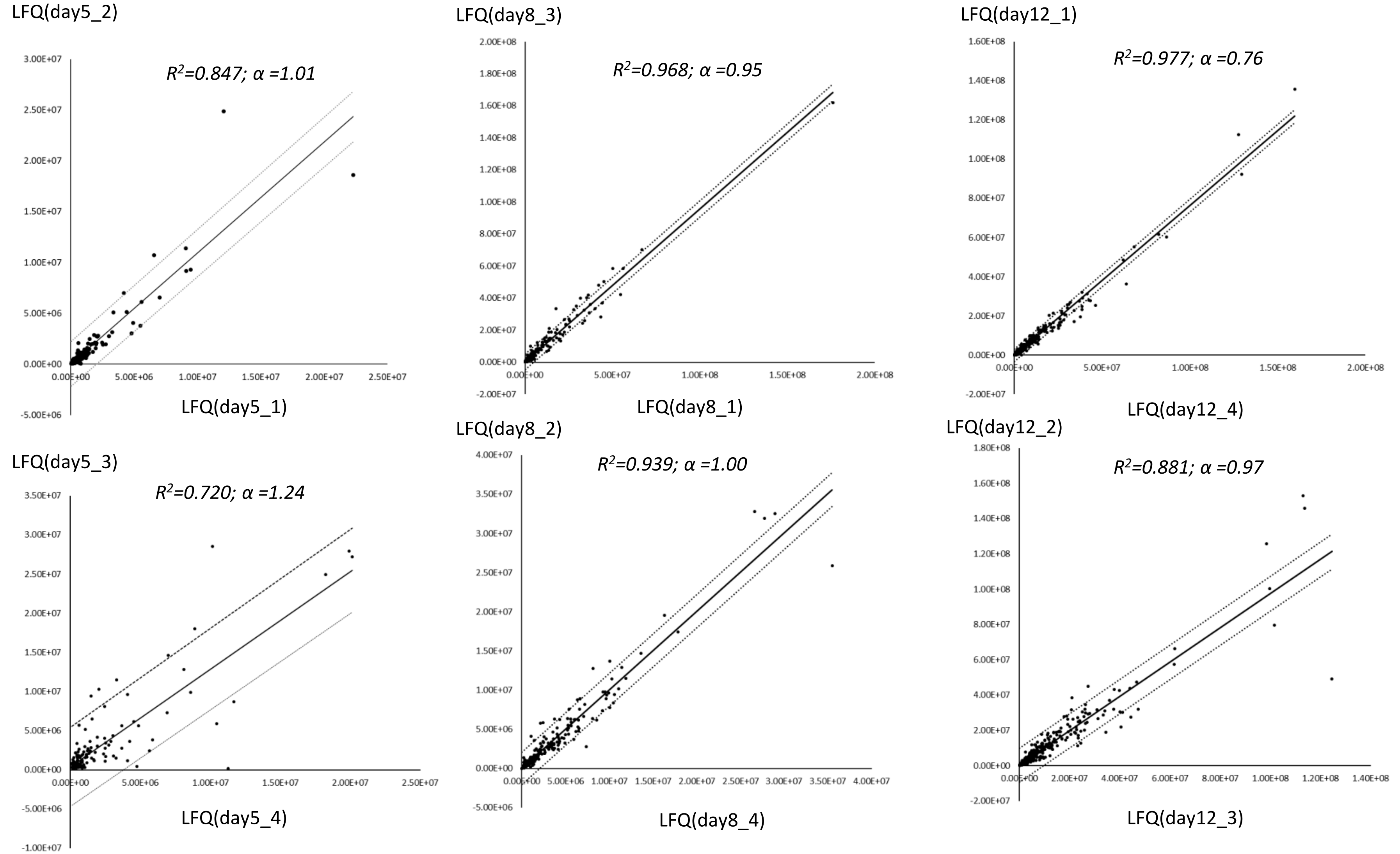

Figure S1.
Reproducibility of quantitative data. A) Correlation matrices were calculated on biological replicates (1-4) at each time point. B) Pairs of replicates with the highest linear correlations were normalized on the replicate with the higher total abundance and linear regression analysis was performed (ANOVA test, confidence interval 95%, tolerance 0.0001).

Figure S2

Protein abundances per functional categories. A) Identified proteins were assigned to functional categories and the abundance of each category per time point was calculated as the sum of the abundances of protein components. Stacked-bar charts were constructed for each functional category. The bar colors indicate proteins that start to be detected at day (d)5 (light grey), protein that start to be detected at d8 (green) and proteins exclusively detected at d12 (red). B) General functional categories were subdivided in distinct processes and for each of them was calculated the relative abundance at the different time points (shown as stacked-bar charts).

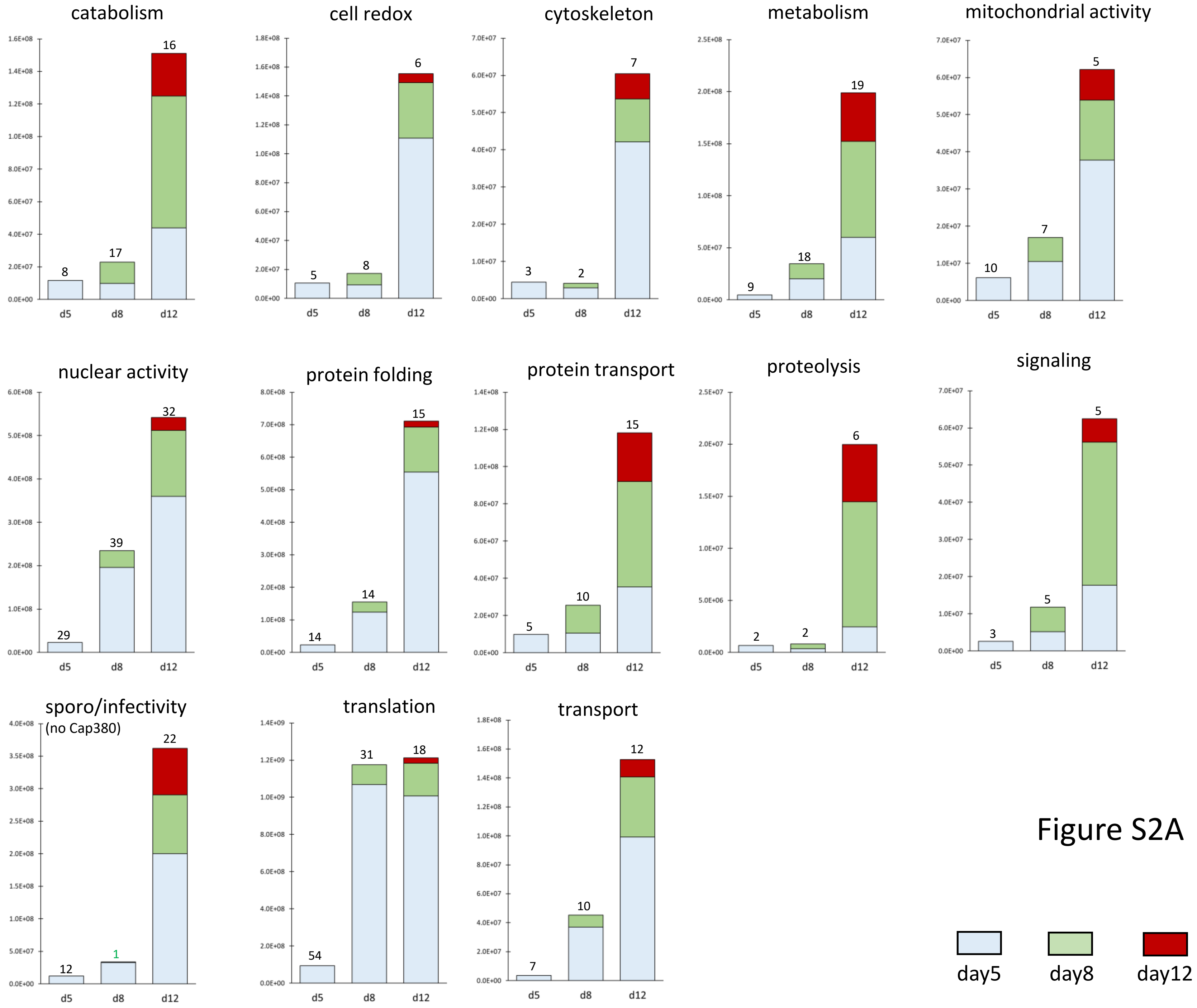

Figure S2A

FigureS2B

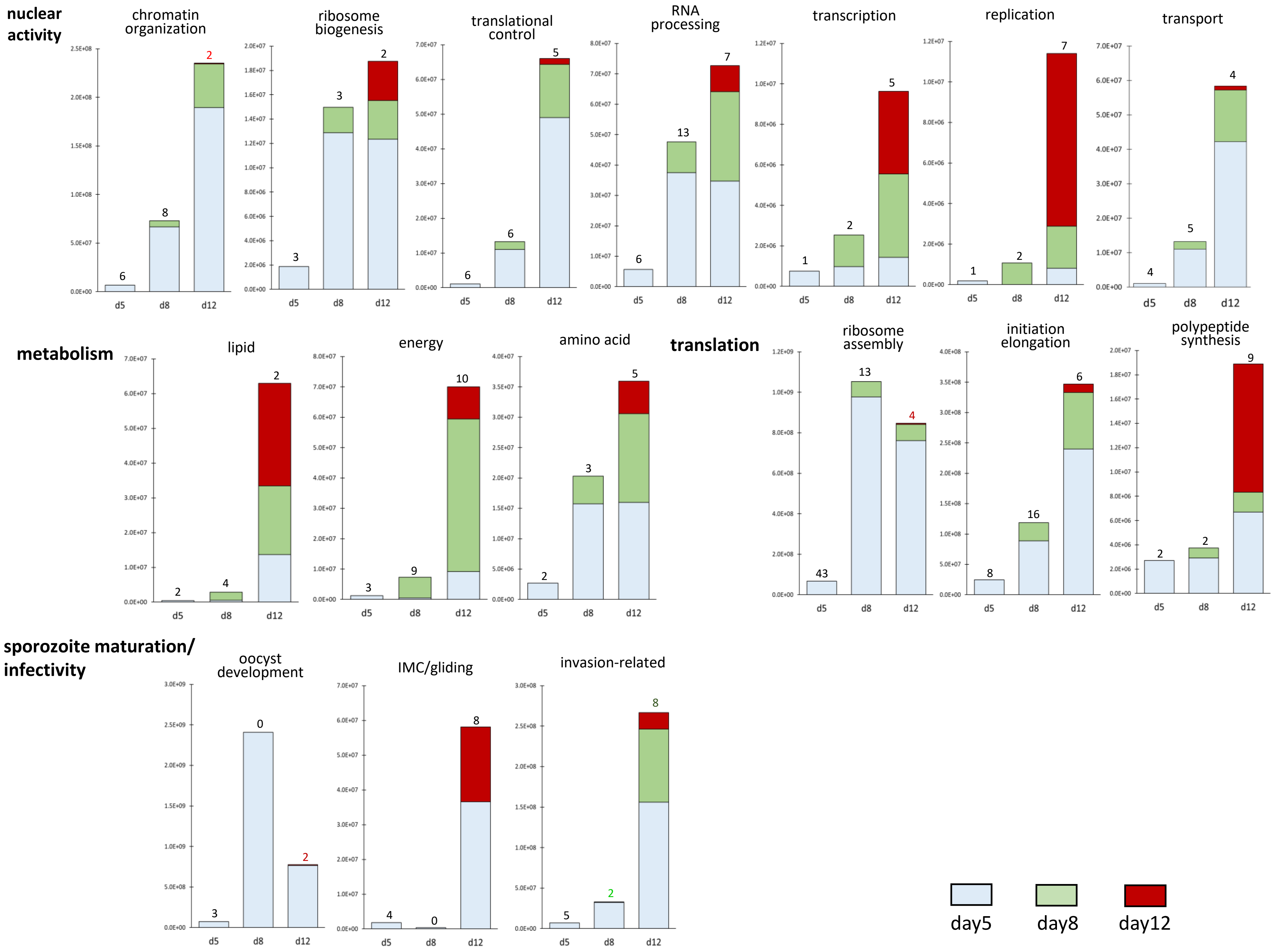
nuclear activity
chromatin organization
ribosome biogenesis
translational control
RNA processing
transcription
replication
transport
metabolism
lipid
energy
amino acid
translation
ribosome assembly
initiation elongation
polypeptide synthesis
sporozoite maturation/ infectivity
oocyst development
IMC/gliding
invasion-related
d5
d8
d12
day5
day8
day12

Figure S3

annotation	tot prot	TM	SP	TM+SP
catabolism	41	1	0	0
cell redox homeostasis	19	6	3	2
cytoskeleton organization	12	0	0	0
metabolism	46	8	4	0
mitochondrial activity	24	9	0	0
nuclear activity	100	1	0	0
protein folding	43	6	9	2
protein transport	30	8	1	0
proteolysis	10	1	2	0
signaling	13	1	1	1
sporo maturation/infectivity	38	4	11	9
translation	103	2	2	0
transport	29	14	0	1
unknown	60	16	5	6

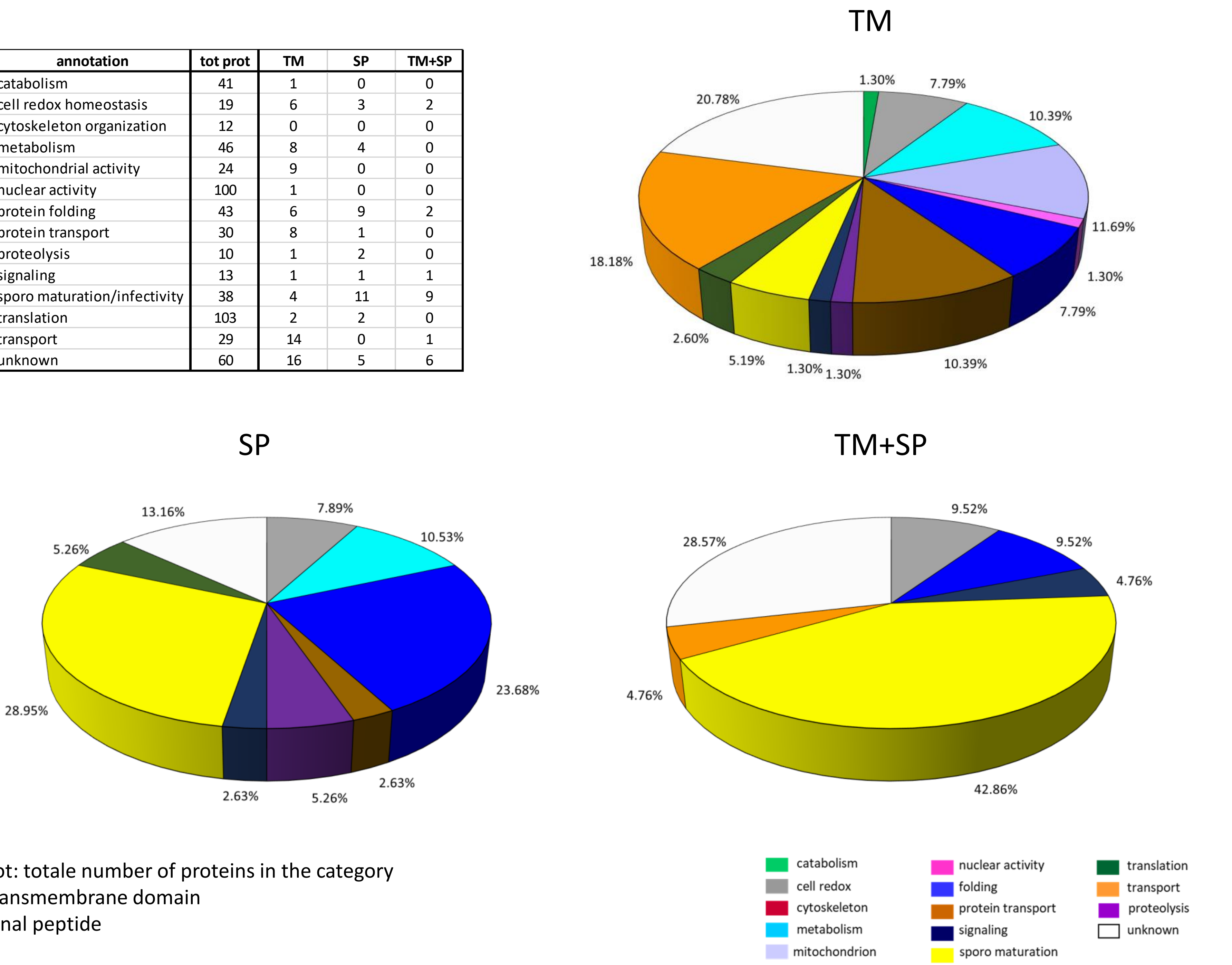

tot prot: totale number of proteins in the category
TM: transmembrane domain
SP: signal peptide

Figure S4

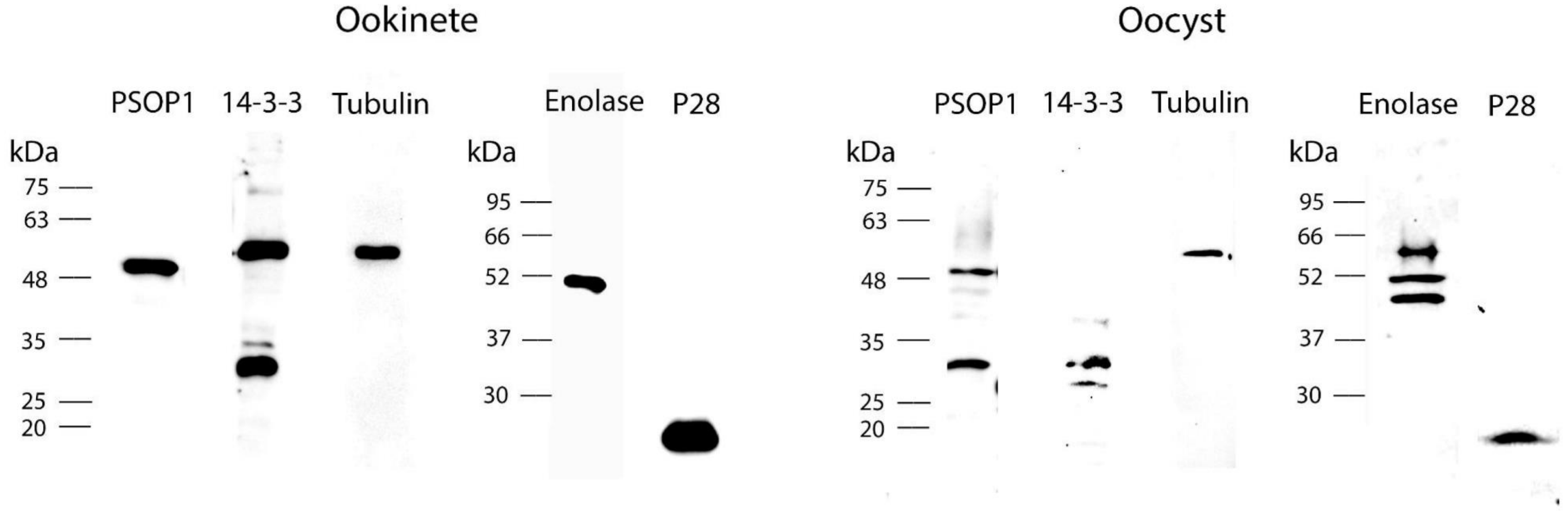

Figure S4. Western blot analysis on ookinete and mature oocyst samples using selected antibodies to confirm protein detection. A) Selected antibodies to detect PSOP1, 14-3-3, beta-tubulin, Enolase and P28 proteins were tested in ookinetes, and each antibody recognizes the antigen at the right size. B) Same antibodies were tested on mature oocyst sample, showing that all are recognizing the right antigen on the membrane.
