# Supplementary material for: A Time Point Proteomic Analysis Reveals Protein Dynamics of Plasmodium Oocysts
Source: Mol Cell Proteomics. 2024 Feb 10;23(3):100736. doi: 10.1016/j.mcpro.2024.100736 (PMC10924140; doi:10.1016/j.mcpro.2024.100736)
Supplement: Supplemental Tables [file mmc2.docx]

Table S1. Mass spectrometry data. For each identified protein the list of corresponding parameters is reported as well as the abundance values in each of the four replicates at day 5, day 8 and day 12.

Table S2. Proteins identified by quantitative mass spectrometry. For the 581 proteins identified by mass spectrometry the corresponding *P. falciparum* orthologue is reported with proteomic evidence obtained for *P. berghei* and *P. falciparum*. The gene name and protein description are reported with proposed functional annotation based on GO terms from PlasmoDB. Based on the protein-protein interaction network obtained from SMART database, the cluster to which belongs the protein is indicated (PPI clusters) as well as the cluster defined according to the hierarchical clustering (expression clusters) derived for abundance profiles (absolute abundances are also reported). Information related to transmembrane domains (TM) and the presence of signal peptide (SP) are also shown. Antibodies used for experimental validation are in the first column. According to proteomic evidence proteins specific of sporozoite stage (green boxes), identified in both erythrocyte invasive stages and sporozoites (orange boxes), exclusively identified in oocyst (pink boxes) are highlighted.
